# Incidence, pattern and prognosis of brain metastases in patients with metastatic triple negative breast cancer

**DOI:** 10.1186/s12885-018-4371-0

**Published:** 2018-04-19

**Authors:** Jia Jin, Yu Gao, Jian Zhang, Leiping Wang, Biyun Wang, Jun Cao, Zhimin Shao, Zhonghua Wang

**Affiliations:** 1 Department of Medical Oncology, Fudan University Shanghai Cancer Center, Shanghai, 200032 People’s Republic of China; 2 Department of Oncology, Shanghai Medical College, Fudan University, Shanghai, People’s Republic of China; 3 Department of Breast Surgery, Fudan University Shanghai Cancer Center, Shanghai, People’s Republic of China; 4 Shanghai, People’s Republic of China

**Keywords:** Triple-negative breast cancer, Brain metastases, Prognosis, Recurrence pattern

## Abstract

**Background:**

To identify the incidence, recurrence pattern and prognosis of brain metastases (BM) among women with metastatic triple negative breast cancer (mTNBC) treated consecutively at a single institution during a 7-year period.

**Methods:**

Patients with histologically confirmed mTNBC were retrospectively identified. The incidence of BM as first site of recurrence and the cumulative BM incidence were computed. We used the Cox proportional hazards model to identify the univariate and multivariate factors associated with survival.

**Results:**

Four hundred thirty three patients were included with a median overall survival (OS) of 21.6 months after median follow-up for 48.1 months. BM was found in 29% (127/433) of the patients and about a quarter (32/127) of BM was first recurrence. The cumulative incidence of BM at 1 and 2 years was 17 and 25%, respectively. The median time from the diagnosis of extracranial metastases to BM was 10 months. Median OS following a diagnosis of BM was 7.3 months. The longer median OS from time of first recurrent BM was noted compared with those of subsequent recurrent (17.3 vs 6.3 months, *p* = 0.008). However, patients with first recurrent BM were associated with shorter OS compared with those without BM (17.3 vs 22.1 months, *p* = 0.006). The independent factors that increased BM death risk were > 3 brain lesions, no BM-directed treatment, subsequent recurrent BM, symptomatic BM and uncontrolled extracranial metastasis.

**Conclusions:**

Patients with mTNBC have a high incidence of early BM with subsequent poor survival. The findings lend support to consideration of screening imaging of the brain for mTNBC patients.

## Background

Breast cancer is the most frequently diagnosed tumor and the second leading mortality in female world [1]. It is also the second most common solid malignancy to metastasize to the brain, estimated to be present at the time of diagnosis of breast cancer in 0.41% of patients, constituting 7.56% of all metastatic sites [2, 3]. Another case series had reported the estimated incidence of brain metastasis (BM) patients with metastatic breast cancer (MBC) ranged from 10 to 16% [4]. The development of BM remains one of the intractable problem for patients with MBC that results in poor morbidity and high mortality. Neurological impairments affected both cognitive and sensory functions and after the diagnosis of BM, the mortality within 1 year was about 80% [4, 5].

The risk of BM has been shown to correlate with breast cancer subtype, and patients with triple negative or Human epidermal growth factor receptor-2 (HER2)-positive subtypes experience significantly higher BM occurrence than patients with luminal-like disease [6–10], having a 3.5–3.6 fold increased risk compared with that of luminal tumors [2, 9, 11]. A study from Dana-Farber Cancer Institute of 116 metastatic triple negative breast cancer (mTNBC) had an increased probability of BM with an estimated risk as high as 46% prior to death [12]. Morever, prognosis after BM occurrence is also subtype-dependent. If patients with triple negative breast cancer (TNBC) develop recurrence, the subsequent survival is poor. TNBC patients had a strongly shorter median survival after BM than in the HER2- positive subtype [13–16]. Identification of biological and prognostic features associated with mTNBC, and development of effective therapeutic strategies for this aggressive subtype of breast cancer are needed.

We aimed to calculate the incidence of BM, to describe the recurrence pattern of BM, to analyse the outcomes after BM relapse and define the implications for prognostic factors of mTNBC patients in a large cohort at one single institution.

## Methods

Patient data at Fudan University Shanghai Cancer Center were collected with the approval of the institution review board and were maintained in a confidential manner.

Medical records ranging from Jan 1, 2010 to Dec 31, 2016, covering a 7-year span, was included for review and extraction based on the following criteria: Patients with histologically confirmed mTNBC, documented based on immunohistochemistry (IHC) with estrogen receptor (ER) negative (IHC < 1%), progesterone receptor (PgR) negative (IHC < 1%), and HER2 negative. HER2 status was assessed by IHC and/or fluorescence in situ hybridization (FISH). HER2 negative was defined as no staining by IHC, and HER2 gene amplification by FISH was performed for those cases of 2+ by IHC and confirmed absence of gene amplification. Main exclusion criteria were bilateral breast cancer, other invasive malignant diseases within the past 5 years except excised basal cell skin carcinoma and cervical carcinoma in situ [5].

All patient identifiers were removed from the dataset, and no personal information on any patient or treating physician could be obtained. All the information available in the dataset was used exclusively for the purpose of this study and was not shared. Two investigators reviewed and extracted all relevant data independently, using standardized data extraction forms. Clinical characteristics, pathologic characteristics, imaging studies, treatment methods, and survival information were obtained. Survival data collected included date of death or periodical survival follow-up call per hospital routine or requirement of clinical trials. Disagreements were resolved by discussion with an independent expert.

The date of diagnosis of BM was based on the radiologic scan date. Overall survival (OS) was calculated from the date of diagnosis of mTNBC to date of death from any cause or last follow-up. Survival subsequent to the development of BM was measured from the date of BM to date of death from any cause or last follow-up. All patients alive at the time of the analysis were censored using the date of last follow-up.

All data was analyzed retrospectively. The Kaplan-Meier method was used for survival analysis and the cumulative BM incidence at 1 and 2 years. Differences between the Kaplan-Meier curves were evaluated using the log-rank test. Actuarial curves were compared by the two-tailed log-rank test and difference of *p* < 0.05 was considered significant. Univariate and multivariate factors associated with survival were analyzed using the Cox proportional hazards model. The estimates of the models are given as hazard ratio (HR) with 95% confidence intervals (95% CI). All statistic analyses were performed using SPSS 17.0 software (SPSS Inc., Chicago, IL, USA).

## Results

### Patient and tumor characteristics

A total of 433 mTNBC patients who were admitted to our hospital consecutively between Jan 1, 2010 to Dec 31, 2016 were identified. The median follow-up time of this study was 48.1 months (range, 5.7–78.7 months). General characteristics of these patients are summarized in Table 1. Median age at diagnosis of mTNBC was 48 years old (range: 23–78 years), with 97 (22%) patients ≤40 years, while 286 (66%) were pre- or perimenopause. The most common pathology were invasive ductal carcinoma (97%) and grade III disease (77%). Three hundred eighty one patients (88%) had non-metastatic primary breast cancer who later developed metastatic disease while 52 (12%) presented with stage IV mTNBC at initial diagnosis. Among early stage breast cancer, 351(92%) of the patients received neo/adjuvant chemotherapy, 323 (85%) had received anthracycline-containing regimen; 268 (70%) had received taxanes and anthracycline neo/adjuvant regimen. After the diagnosis of breast cancer, 114 (26%) patients developed recurrence with one year. Thirty-seven (9%) had ≥3 metastatic organ sites while visceral involvement was noted in 252 (58%) patients.

**Table 1:** Patient Characteristics at Baseline (*N* = 433)

Characteristics	No.	Percentage (%)
Age, years		
Median (Range)	48 (23–78)	
≤ 40	97	22.2
> 40	336	77.8
Menopausal status		
Pre- or perimenopause	286	66.0
Postmenopause	138	31.9
Unknown	9	2.1
Histological subtype		
Invasive ductal	419	96.8
Lobular	6	1.4
Metaplastic	6	1.4
Medullary	2	0.5
Grade		
I-II	100	23.1
III	333	76.9
DFI		
≤ 1 year	114	26.3
> 1 year	267	61.7
IV	52	12.0
Prior neo/adjuvant chemotherapy		
Anthracycline-containing regimen	323	84.8
Anthracycline and Taxanes regimen	268	70.3
Cyclophosphamide	340	89.2
Initial site of mTNBC		
Lymph nodes	247	57
Liver	101	23.3
Lung	183	42.3
Bone	105	24.3
Pleural effusion	52	12
Local recurrence	105	24.2
Brain	32	7.4
Contralateral breast	2	0.5
Visceral metastasis		
Yes	252	58.2
No	181	41.8
Number of metastatic organ sites		
≥ 3	37	8.5
< 3	396	91.5

*DFI* disease free interval

### Development of brain metastases in patients with mTNBC

29% (127/433) of the patients developed BM, among whom about a quarter (32/127) presented BM at initial diagnosis of mTNBC. About half (14/32) of patients in the “first recurrent BM group” had synchronous extracranial metastases. The most common involved sites were lymph nodes (*n* = 14), lung (*n* = 11), bone (*n* = 8), and liver (*n* = 5). The cumulative incidence of BM at 1 and 2 years was 17% and 25%, respectively. The median time from the diagnosis of extracranial metastases to BM was 10 months (95% CI, 8.7–11.4).

Seventy-six patients (60%) had symptomatic brain metastasis. The most common symptoms of BM were headache (40%), vomiting (23%), motor impairment (12%), and vertigo (7%).

Sixty-nine (54%) patients had three or fewer brain lesions, and 58 (46%) had more than three lesions. Intracranial metastases were located in the supratentorial region in 65 patients (51%), in the infratentorial region in 12 patients (9%), in both supra-and infratentorial regions in 30 (24%), in the brainstem in 6 patients (5%), and 14 patients (11%) had meninges involvement. Sixty-nine patients (54%) had limited intracranial metastases (number of metastases ≤3).

### Survival following brain metastases and prognostic factors

**Fig. 1:**
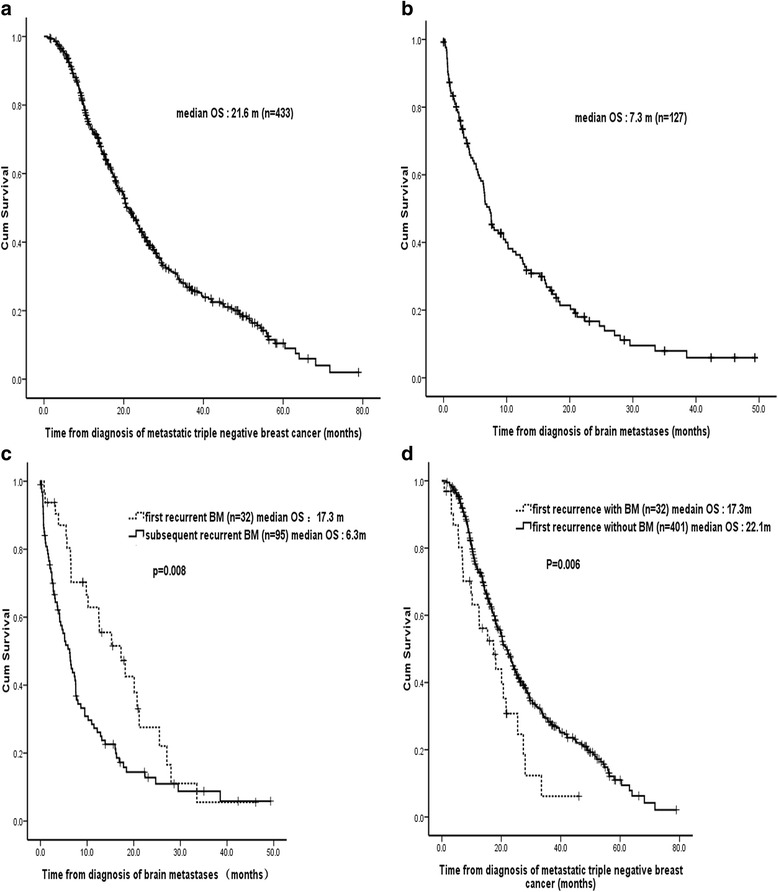
Overall survival of patients with mTNBC. **a** Kaplan–Meier survival curve of 433 patients with mTNBC. **b** Kaplan–Meier survival curve of 127 patients with BM from TNBC. **c** Kaplan-Meier plots illustrating survival of patients had BM initially involved and those subsequently involved. The longer median survival from time of brain metastases diagnosis that is seen in the first recurrent BM compared with the subsequent recurrent BM (17.3 vs 6.3 months, *p* = 0.008). **d** Kaplan-Meier plots illustrating survival of patients with first BM recurrence presence and those absence. Patients with BM initial presence were associated with shorter OS (17.3 vs 22.1 months, *p* = 0.006)

At the time of the analyses, 298 (69%) patients had died and OS at 1 and 2 years was 72 and 44%, respectively. For the total 433 mTNBC patients, median OS was 21.6 months (95% CI 19.5–23.7) (Fig. 1a). Among the 127 patients who developed BM, 103 (81%) had died. Initial treatment was whole-brain radiation therapy (WBRT) in 74 patients (58%), stereotactic radiosurgery (SRS) in 7 patients (6%), WBRT and SRS in 8 patients (6%), surgical resection in 9 patients (7%) and 29 patients (23%) did not receive any treatment for BM. A total of 16 (50%) patients received platinum, 16 (50%) patients received gemcitabine, 8 (25%) patients received taxanes, 10 (31.2%) patients received capecitabine and 1 (3%) patient received bevacizumab as part of the treatment of mTNBC in the “first recurrent BM group”. And the patients with first recurrence without BM had more chances to subsequent chemotherapies. The proportion of patients received platinum, gemcitabine, taxanes, capecitabine and bevacizumab was 288 (71.8%), 342 (85.3%), 233 (58.1%), 175 (43.6%) and 64 (16.0%) patients, respectively.

Thirty-seven patients (36%) died from intracranial disease. Twenty-nine patients (28%) died due to progression of extracranial disease and their BM were controlled at the time of death. Thirty-six patients (35%) died from progression of both intracranial and extracranial disease. One patient died from sepsis.

Median survival following the occurence of BM was 7.3 months (95% CI, 6.1–8.4) (Fig. 1b) and OS at 1 and 2 years was 36 and 15%, respectively. Among the 32 patients who developed BM at mTNBC presentation, 23 have died. Among other 401 patients whose first metastasis was extracranial metastases, 275 have died. Median OS from time of BM as first recurrence was longer compared with subsequent recurrence (17.3 vs 6.3 months, *p* = 0.008) (Fig. 1c). However, patients with first recurrent BM were associated with shorter OS (17.3 months, 95% CI 9.0–25.7) compared with those without first BM recurrence absence (22.1 months, 95% CI 19.9–24.3) (*p* = 0.006) (Fig. 1d).

Univariate and multivariate regression analyses identified prognostic factors for survival (Table 2). The independent factors that increased BM death risk were > 3 brain metastasis (HR: 2.37, 95% CI: 1.47–3.80; *p* = 0.001), no BM-directed treatment (HR: 2.30, 95% CI: 1.32–3.92; *p* = 0.002), BM as the subsequently involved (HR: 2.21, 95% CI: 1.18–4.17; *p* = 0.01), symptomatic brain metastasis (HR: 1.92, 95% CI: 1.13–3.27; *p* = 0.01) and uncontrolled extracranial metastasis (HR: 1.78, 95% CI: 1.13–2.92; *p* = 0.02).

**Table 2:** Univariate and multivariate models for OS with BM in mTNBC patients (*n* = 127)

		Univariate			Multivariate		
Factor	No.	Median	95% CI	*P*	HR	95% CI	*P*
Age				0.55			
≤ 40	29	6.4	1.7–11.1				
> 40	98	7.3	6.1–8.5				
DFI				0.33			
≤ 1 y	31	7.6	4.7–10.5				
> 1 y	76	7.6	4.5–11.7				
IV	20	5.4					
KPS				0.000			0.07
70–100	109	7.6	4.7–10.4		1.81		
40–60	18	1.7	0.2–3.4				
Recurrent BM				0.008			0.01
First	32	17.3	9.2–25.3		2.21	1.18–4.17	
Subsequent	95	6.3	4.5–8.0				
Number of intracranial metastases				0.002			0.001
> 3	58	5.4	2.1–8.7		2.37	1.47–3.80	
≤ 3	69	10.2	6.3–14.1				
Symptomatic brain metastasis				0.02			0.01
Presence	76	5.5	2.8–8.2		1.92	1.13–3.27	
Absence	51	8.7	3.0–16.7				
Meninges involvement				0.01			0.48
Yes	14	3.2	0.9–5.4		1.32	0.61–2.83	
No	113	7.6	4.8–10.3				
Brain-directed treatment				0.001			0.002
Not performed	29	3.7	0.2–7.7		2.30	1.32–3.92	
Performed	98	9.5	6.3–12.7				
Extracranial metastasis^a^				0.002			0.02
Under-control	37	9.8	5.8–13.8		1.78	1.13–2.92	
Out-of-control	65	3.6	2.2–4.9				

^a^103 patients died

## Discussion

In this study, we describe the incidence of BM among 433 patients with mTNBC and characterize the subsequent survival of such patients. BM was found in 29% of patients prior to death and about a quarter of BM was first recurrent at the time of diagnosis of metastatic disease. Median survival time following a diagnosis of BM was 7.3 months and patients with BM who were asymptomatic, with 1–3 metastases, received locoregional treatment, presented as first recurrence or controlled extracranial metastasis were independent predictors of better survival.

Patient-, disease- and treatment-related factors have effects on the prognosis of BM from breast cancer. Our findings are consistent with the modified breast graded prognostic assessment (breast-GPA) [17], based on a prospectively maintained institutional database (*n* = 1552). The authors suggested number of BM (> 3 vs. ≤3) is a strong and independent prognostic factor, besides age, tumor subtype, and Karnofsky performance status (KPS). In a study of 1256 patients with BM for breast cancer from 24 member institutions in Japan and a recent study focused on patients with brain-only metastases from breast cancer, several prognostic factors for longer survival were identified in multivariate regression analysis, including asymptomatic brain disease and active treatment of BM [10, 18]. In addition to molecular subtype, the cumulative number of extracranial lesions is another risk factor for BM development among patients with metastatic disease. The risk of BM is significantly higher in patients with metastatic disease that involved more than 3 extracranial sites, including bone, lung, and liver (vs 0 or 1 site; odds ratio, 3.40; *p* < 0.001) [3]. In early breast cancer setting, historical data suggest risk factors for BM included young age, lymph node positive, grade 3, TNBC or HER2 positive.

TNBC typically carried increased risk for early distant metastases, higher incidence of lung and brain involvement, and overall poorer survival outcome, as more characteristics of TNBC had been demonstrated and validated. Our findings are consistent with previous data indicating a higher propensity of approximately 30 to 46% mTNBC patients will eventually develop BM prior to death [19, 20], while the frequency was about 10% in luminal tumors [12, 21]. In addition, TNBC is also associated with higher risk (3.5 - 4.7%) of developing BM as first site of recurrence, compared with non-selected breast cancer patients (1.3%) [22, 23]. This may be the result of the inherent aggressiveness of TNBC, the predominance of infiltrative basal-like type. And because systemic chemotherapy does not adequately cross the blood brain barrier, the brain can be a sanctuary [24].

This current study showed that a high proportion of TNBC patients (up to 7.4%) are diagnosed with BM at the time of initial metastatic diagnosis, and was consistent with a previous report. Several studies reported TNBC showed the shortest interval from primary early breast cancer to development of BM. Heitz F et al. [7] previously reported that shortest interval for triple-negative patients (22 months), and longer intervals for HER2-positive (30 months) and ER+/HER2- (63.5 months) breast cancer. In larger sample size, our results are further validated by previous studies that patients with BM development in TNBC subtype displayed a dismal median survival of 3.7–7.2 months [2, 10, 13, 15, 18, 22, 24], whereas HER2-positive and HR+/HER2 − subtypes displayed median survival 16.5–27.9 months and 9.3–14.0 months [2, 25, 26], respectively.

One notable finding was, however, in contrary to previous reports stating that patients with BM at initial diagnosis carried poor prognosis (Dawood et al. [22] reported only 5.8 months), we reported for the first time that the median survival for patients with first recurrent BM (*n* = 32), was significantly longer than those with subsequent BM (*n* = 95), 17.3 months vs 6.3 months, *p* = 0.008. The potential causes are analyzed as follows. First, patients with BM as the first site of metastasis may have good KPS. On the contrary, the presence of extracranial disease of subsequent BM may play an important part in the whole disease. Because extracranial disease may have implications on organ function and choice of systemic therapy. Second, in our study, 14 (44%) of 32 patients with first recurrent BM were found to be asymptomatic due to screening for potential clinical trials that were conducted at our institution. When BM were identified early, they were typically amenable to potentially radical therapy. In our study, 26 (81%) of 32 patients had received local treatment for BM. The treatment between the screening group and the “symptomatic first recurrent BM group” did not differ significantly, and about half of each group underwent SRS. Patients in the screening group were associated with longer OS compared with the “symptomatic first recurrent BM group”, but with no statistical difference (*p* = 0.516). This difference in survival may be under estimated, given our small sample size and subsequent limited power to calculate the difference in survival. Although current breast cancer screening guidelines do not recommend routine assessment of BM in patients with metastatic disease, our results support consideration of screening for BM in mTNBC patients given the high incidence of BM in TNBC patients with extracranial disease.

Moreover, our result is consistent with previous studies and support the notion that the natural course of BM is also strongly influenced by the biology of the breast cancer subtype. The cause of death in mTNBC patients with BM is rarely due to progressive intracranial lesions alone, in our study 29% (37/127), in contrast to HER2-positive breast cancer patients with BM, a setting in which up to 50% of patients die of progressive BM disease [12]. Patients had a prolonged disease control of extracranial disease due to the advancement of active anti-HER2 treatments. Lin NU et al. [12] reported a group of 42 patients treated for BM from mTNBC at Dana-Farber Cancer Institute, only 3 patients were judged to have stable or responsive systemic disease in the face of progressive intracranial disease at the last follow up prior to death. Of the 42 patients, 12 (28.6%) died primarily of systemic disease progression, 17 (40.5%) died of both systemic and BM progression and only 11 (26.2%) died primarily of BM progression. Morris et al. [27] found that following diagnosis of BM, 91% of patients had evidence of progression of the extracranial disease during the course of their disease in their study. Both extracranial disease and BM contribute to the poor survival outcome for mTNBC with BM patients. Since most of the patients with BM from TNBC will have systemic disease progression prior to death, there is an urgent need to develop therapies that are effective in systemic therapy rather than close attention to BM alone.

Due to the lack of prospective studies exclusive to BM in TNBC, there is no specific treatment guidelines for its management. In daily practice, the number of brain lesions, the availability of systemic treatment options, and the presence or absence of extracranial metastases are considered. WBRT has been a mainstay of treatment for several decades [28, 29]. Prolonged survival and better neurocognitive function preservation are due to the high response rate of 60% after WBRT [30].

However, there are some notable long-term risks of serious and permanent toxic effects, such as cerebellar dysfunction and cognitive deterioration. Nowadays, local therapeutic options as initial treatment are being used with increasing frequency, in order to minimize potential long-term morbidity following WBRT. With tumor number of BM less than four, aggressive local treatments involving either surgical resection or SRS may be applied, with potential curative intentions [31, 32]. The immediate relief of intracranial hypertension and improvement of focal deficits are the additional advantages of surgery and SRS. Nowadays, a local tumor control rate at 1 year of 80–90% with median survival of 6–12 months has been reported for SRS [30, 33]. Therefore, the number of BM and the treatment options have significant effect on the survival. In this study, median survival from BM in patients with and without brain-directed treatment was 9.5 and 3.7 months, respectively (*p* = 0.001). Median survival of patients underwent WBRT, SRS, WBRT+SRS, surgery and no brain-directed treatment was 8.4, 12.9, 9.3, 11.2 and 3.7 months, respectively. Kaplan-Meier survival analysis indicated that patients with WBRT-based treatment (*n* = 82) exhibited longer OS than patients without WBRT (*n* = 45) (8.7 vs 5.4 months, *p* = 0.019). Because the numbers were too small, the survival between the subgroup of SRS, SRS+ WBRT, or surgery did not differ significantly.

There are some limitations regarding this study. First, this study was conducted in a single, academic medical center, there might be some referral bias. Second, consistent with clinical practice, triple-negative phenotype was diagnosed on the primary breast tumor and no further re-biopsy of metastatic lesions was performed in the majority of the cases. Therefore, discordance in hormone receptor and HER2 status between primary and metastatic lesions could not be ruled out. To draw a complete picture of this neglected group, prospective studies specifically designed to measure these endpoints other than overall survival are still needed.

## Conclusions

Patients with mTNBC have a high incidence of early BM with subsequent poor survival. In conclusion, asymptomatic, limited number of metastases, receive any locoregional treatment, first recurrent BM or controlled extracranial metastasis were independent predictors of better survival for mTNBC with BM. The findings lend support to consideration of screening imaging of the brain for mTNBC patients.

## Abbreviations

BM: Brain metastases
CI: Confidence intervals
DFI: Disease free interval
ER: Estrogen receptor
FISH: Fluorescence in situ hybridization
GPA: Graded prognostic assessment
HER2: Human epidermal growth factor receptor-2
HR: Hazard ratio
IHC: Immunohistochemistry
KPS: Karnofsky performance status
MBC: Metastatic breast cancer
mTNBC: Metastatic Triple negative breast cancer
OS: Overall survival
PgR: Progesterone receptor
SRS: Stereotactic radiosurgery
WBRT: Whole-brain radiation therapy
